# Visual Cortex Engagement in Retinitis Pigmentosa

**DOI:** 10.3390/ijms22179412

**Published:** 2021-08-30

**Authors:** Gianluca Pietra, Tiziana Bonifacino, Davide Talamonti, Giambattista Bonanno, Alessandro Sale, Lucia Galli, Laura Baroncelli

**Affiliations:** 1 Neuroscience Institute, National Research Council (CNR), I-56124 Pisa, Italy; pietrag84@gmail.com (G.P.); davidetalamonti29@gmail.com (D.T.); sale@in.cnr.it (A.S.); galli@in.cnr.it (L.G.); 2 Section of Pharmacology and Toxicology Unit, Department of Pharmacy, University of Genova, I-16148 Genova, Italy; tiziana.bonifacino@gmail.com (T.B.); bonanno@difar.unige.it (G.B.); 3 Department of Life Science, University of Trieste, I-34128 Trieste, Italy; 4 IRCCS Ospedale Policlinico San Martino, I-16132 Genova, Italy; 5 Department of Developmental Neuroscience, IRCCS Stella Maris Foundation, I-56128 Pisa, Italy

**Keywords:** retinitis pigmentosa, visual cortex, plasticity, inhibition, rd10 mouse model

## Abstract

Retinitis pigmentosa (RP) is a family of inherited disorders caused by the progressive degeneration of retinal photoreceptors. There is no cure for RP, but recent research advances have provided promising results from many clinical trials. All these therapeutic strategies are focused on preserving existing photoreceptors or substituting light-responsive elements. Vision recovery, however, strongly relies on the anatomical and functional integrity of the visual system beyond photoreceptors. Although the retinal structure and optic pathway are substantially preserved at least in early stages of RP, studies describing the visual cortex status are missing. Using a well-established mouse model of RP, we analyzed the response of visual cortical circuits to the progressive degeneration of photoreceptors. We demonstrated that the visual cortex goes through a transient and previously undescribed alteration in the local excitation/inhibition balance, with a net shift towards increased intracortical inhibition leading to improved filtering and decoding of corrupted visual inputs. These results suggest a compensatory action of the visual cortex that increases the range of residual visual sensitivity in RP.

## 1. Introduction

Retinitis pigmentosa (RP) is a group of rare, inherited disorders involving the progressive breakdown and loss of retinal photoreceptors. Genetic mutations responsible for RP produce biochemical defects in multiple pathways, but the common result is the degeneration of rod and cone photoreceptors, which causes loss of night vision, gradual narrowing of the visual field, dyschromatopsia, and eventually a decline in visual acuity [1,2]. This retinopathy affects roughly 1 in 4000–5000 people worldwide [3]. While there are no effective treatments for RP, recent research is focusing on novel therapeutic strategies, including pharmacological targeting, gene augmentation therapy, cell transplants, and electronic prosthesis, aimed at preventing photoreceptor degeneration or replacing light-responsive parts of the retina [4,5,6,7,8,9,10].

Although these avenues are promising for vision recovery and/or preservation in RP patients [2], the integrity of the visual system beyond the photoreceptor layer is a crucial constraint for the success of any retinal treatment. Despite a gradual remodeling of inner retinal neurons and a decline in the signal-to-noise ratio in the output of ganglion cells, anatomical and functional studies have described a global preservation of the retinal structure [11,12,13,14,15,16,17]. Poor light responsiveness and defective signal-to-noise ratio have also been reported in the thalamus and superior colliculus of RP models and patients [18,19]. Moreover, the optic pathway conveying visual signals to the cortex appears mildly harmed [5,20,21,22], and electrical stimulation is able to elicit reliable responses in the visual cortex of RP patients, thereby generating the illusory perception of light flashes [23,24,25,26]. However, the ability to evoke phosphenes is reduced in RP patients, particularly in subjects with a high level of visual deafferentation [23,24,25,26].

In contrast, studies portraying the functional arrangement of the visual cortex in RP are scarce. Although general alterations of brain activity have been described in individuals with RP [27,28,29] and animal models [30,31], the impact of retinal degeneration on cortical circuits is largely unknown. Only recently, it has been shown that the adult brain of RP patients retains a good level of short-term plasticity [32], and we have reported that visual cortical circuits in rd10 mice preserve their capability of input-dependent remodeling until an advanced stage of retinal damage [33]. 

Here, we investigated whether intrinsic processing of cortical circuits changes during the progression of retinal deterioration in rd10 mice [34,35]. We found that the visual cortex undergoes a significant alteration of the intracortical excitation/inhibition balance alongside photoreceptor degeneration and that antagonizing GABAergic neurotransmission further deteriorates visual functions.

## 2. Results

### 2.1. Altered Excitation/Inhibition Balance in the Visual Cortex of rd10 Animals

It is well known that the balance between excitatory and inhibitory neurotransmission is a key regulator of visual cortex plasticity and the brain’s capability to fine-tune to the input salient features [36,37,38,39,40]. To analyze possible alterations in this balance, we measured the levels of major excitatory and inhibitory markers in the visual cortex of rd10 animals at three ages corresponding to an intermediate (P60), advanced (P120), and late (P180) stage of behavioral visual acuity deterioration.

**Figure 1:**
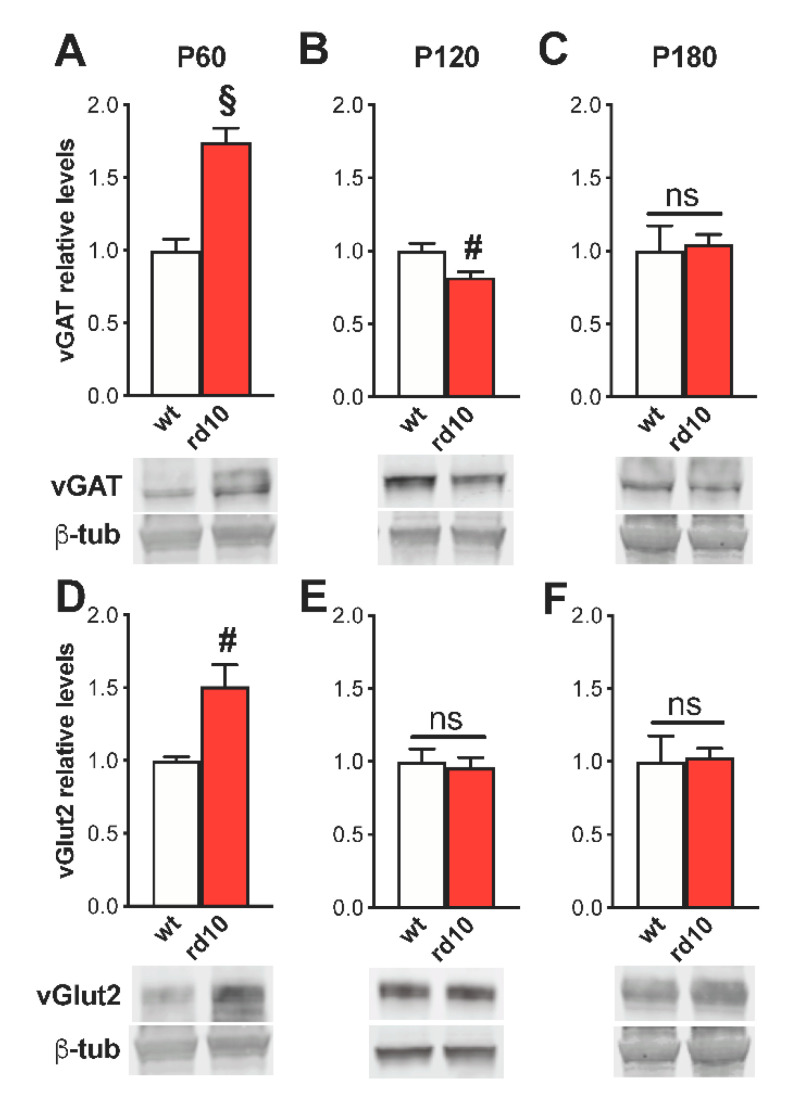
Age-dependent alterations of vGAT and vGlut2 levels in the visual cortex of rd10 animals. In all panels, the inset is a representative immunoblotting showing the corresponding protein levels in the visual cortex of wild-type (wt) and rd10 transgenic animals. β-Tubulin (β-tub) is the internal standard. Diagrams show the percentage value of protein expression in relation to the control (wt) group. (**A**–**D**) At P60, protein levels of vGAT and vGlut2 were almost two-fold increased in the visual cortex of rd10 mice (*n* = 8) compared to age-matched wt mice (*n* = 8; *t*-test, *p* < 0.001 and Mann–Whitney rank sum test, *p* < 0.01). (**B**–**E**) A significant reduction of vGAT protein expression in rd10 mice (*n* = 8) with respect to wt animals (*n* = 8; *t*-test, *p* < 0.01) was present at P120. We did not find a parallel alteration of vGlut2 (*t*-test, *p* = 0.705). (**C**–**F**) At P180, the differences between the two groups were lost (rd10 *n* = 8, wt *n* = 4; *t*-test, vGAT *p* = 0.764 and vGlut2 *p* = 0.863). Data are expressed as mean ± s.e.m. # *p* < 0.01, § *p* < 0.001, and ns: not significant.

We found a time-dependent modulation in the expression of proteins responsible for packaging GABA and glutamate in synaptic vesicles, whereas no alterations were present in the translational regulation of neurotransmitter synthetic enzymes and postsynaptic machinery. At P60, the amount of vGAT, the vesicular transporter of GABA, was strongly increased in the primary visual cortex (V1) of rd10 mice compared to age-matched wt animals (Figure 1A) with a parallel upregulation of the vesicular transporter of glutamate vGlut-2 (mainly expressed in thalamocortical afferents of V1, [41]; Figure 1D). Two months later (P120), protein levels of vGAT were instead reduced in the visual cortex of rd10 mice (Figure 1B), while no alterations in vGlut-2 were present (Figure 1E). By P180, all the differences between rd10 and wt animals disappeared (Figure 1C,F). No discrepancy was detected in Western blot reactivity of the two isoforms of the GABA synthesizing enzyme nor in the intracortical vGlut1 marker at any age tested (Figure S1). In addition, presynaptic alterations were not associated with an imbalance in postsynaptic markers of excitatory versus inhibitory synapses (tested using the scaffolding proteins (postsynaptic density-95 (PSD95) and gephyrin, respectively) (Figure S2).

**Figure 2:**
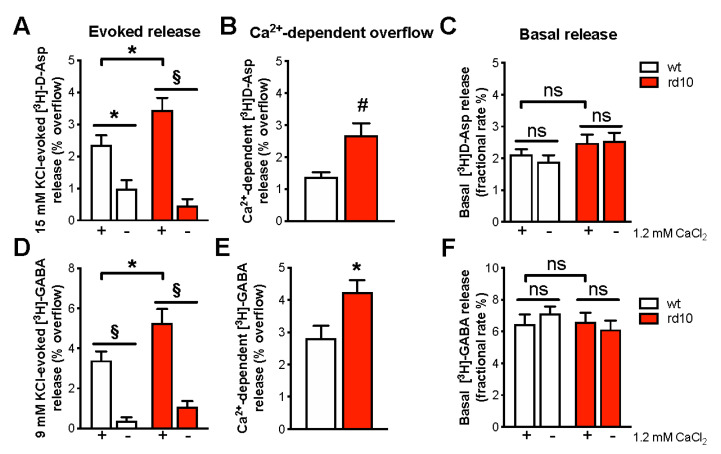
Increased evoked release of glutamate and GABA from synaptosomes of rd10 visual cortex at P60. Synaptosomes were purified from the visual cortex of P60 wt and rd10 mice and incubated in the presence of [^3^H]D-Asp or [^3^H]GABA in order to label the intraterminal releasing pools of glutamate and GABA, respectively. KCl (15 mM for glutamate experiments and 9 mM for GABA experiments) was introduced after 39 min of superfusion. When appropriate, Ca^2+^ was omitted 19 min before KCl (the + symbol below the x axis refers to Ca^2+^-dependent release, the − sign indicates Ca^2+^-free release). Then, samples were counted for radioactivity. Basal release results are expressed as fractional rate percent, while evoked release and Ca^2+^-dependent overflow results are expressed as percent overflow released by high KCl. Data are expressed as mean ± s.e.m. of 4–9 independent experiment samples run in triplicate. (**A**) 15 mM KCl-evoked [^3^H]D-Asp overflow was significantly increased in the visual cortex of rd10 mice (two-way RM ANOVA, post hoc Bonferroni test, *p* < 0.05). As expected, Ca^2+^ removal strongly affected Asp release in both wt (*p* < 0.05) and rd10 animals (*p* < 0.001). (**B**) Ca^2+^-dependent stimulus-evoked [^3^H]D-Asp overflow was larger in rd10 visual cortex (*t*-test, *p* < 0.01). (**C**) [^3^H]D-Asp basal release was not affected by rd10 mutation (two-way RM ANOVA). (**D**) 9 mM KCl-evoked [^3^H]-GABA overflow was significantly higher in the visual cortex of rd10 mice (two-way RM ANOVA, post hoc Bonferroni test, *p* < 0.05). Ca^2+^ removal strongly affected GABA release in both wt and rd10 animals (*p* < 0.001 for both comparisons). (**E**) Ca^2+^-dependent stimulus-evoked [^3^H]-GABA overflow was amplified in the rd10 visual cortex (*t*-test, *p* < 0.05). (**F**) [^3^H]-GABA basal release was not different in rd10 mice compared to wt (two-way RM ANOVA). * *p* < 0.05, # *p* < 0.01, § *p* < 0.001, and ns: not significant.

The dysregulation of presynaptic transporters is likely to influence synaptic release of GABA and glutamate. Thus, we measured spontaneous and KCl-evoked release of [^3^H]D-Asp and [^3^H]GABA in synaptosomes isolated from the visual cortex of wt and rd10 mice. We found that the Ca^2+^-dependent stimulus-evoked overflow of both glutamate (Figure 2A,B) and GABA (Figure 2D,E) was markedly increased in the visual cortex of rd mice with respect to wt animals at P60, indicating a general reinforcement of exocytotic neurotransmitter release. In agreement with Western blot results, at P180, no abnormalities were detected in rd10 animals (Figure S3). Basal neurotransmitter release did not statistically diverge from that of wt mice at any age tested (Figure 2C,F and Figure S3).

These results indicate that retinal degeneration affects cortical levels of excitation and inhibition with potential functional changes in V1 circuits.

### 2.2. Rd Mutation Shifts the Balance towards Inhibitory Overdrive in the Visual Cortex

We recently reported that basal synaptic transmission, assessed by measuring the amplitude of field potentials as a function of stimulus intensity, showed a significantly shallower response in rd10 mice compared to wt animals at P60 [33].

This decreased circuit responsiveness of rd10 mice might stem from three potential changes in physiological activity: (i) diminished intrinsic excitability of cortical pyramidal neurons, (ii) attenuation of the excitatory synaptic drive, or (iii) gain in the inhibitory drive impinging on pyramidal neurons. In a first attempt to discriminate among these possibilities, we assessed the activity and basic properties of synaptic transmission within local cortical microcircuits.

**Figure 3:**
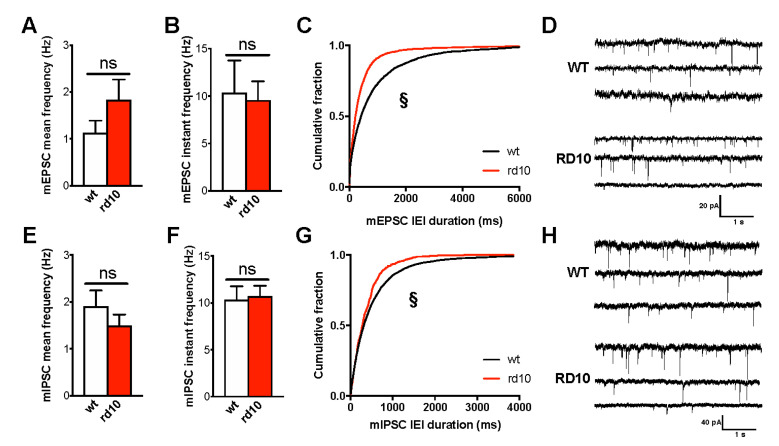
Basal synaptic function in the visual cortex of rd10 mice at P60. (**A**,**B**) Average mEPSC frequency in pyramidal cells of layer II/III of wt (*n* = 11 cells, 6 mice) and rd10 animals (*n* = 10 cells, 7 mice). No difference was found in mean (*t*-test, *p* = 0.174) and instant frequency (Mann–Whitney rank sum test, *p* = 0.504). (**C**) Cumulative distribution of mEPCS interevent intervals highlighted a significant reduction (Kolmogorov–Smirnov test, *p* < 0.001). (**E**,**F**) Average mIPSC frequency in pyramidal cells of layer II/III of wt (*n* = 12 cells, 6 mice) and rd10 animals (*n* = 13 cells, 6 mice). No difference was found in mean (*t*-test, *p* = 0.323) and instant frequency (*t*-test, *p* = 0.834). (**G**) Cumulative distribution of mIPCS interevent intervals showed a small but significant reduction (Kolmogorov–Smirnov test, *p* < 0.001). (**D**–**H**) Representative traces recorded from wt and rd10 neurons voltage clamped at −85 mV for mEPSCs and at −75 mV for mIPSCs. For each group, the middle trace represents the mean frequency, while the top and bottom ones are mean plus and minus SD, respectively. Data are expressed as mean ± s.e.m. § *p* < 0.001, and ns: not significant.

**Figure 4:**
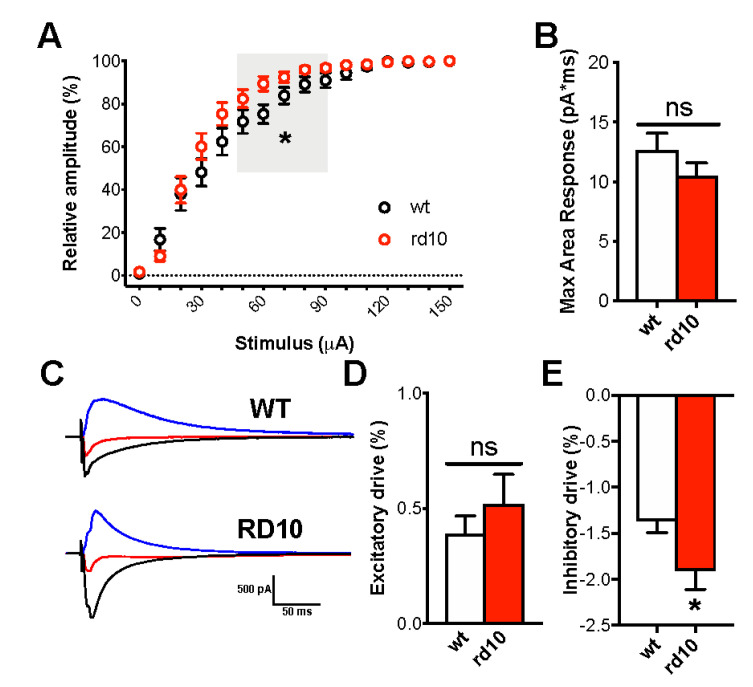
The balance between evoked excitation and inhibition onto pyramidal neurons of layers II/III was altered in rd10 mutants at P60. (**A**) Input–output curves from wt and rd10 single neurons voltage clamped at −82 mV. Electrical stimulation was provided in layer IV. The percentage relative amplitude as a function of stimulus intensity measured in microampere (μA) did show a significant leftward shift in rd10 visual cortex (red circles, *n* = 17 cells, 9 mice) with respect to wt (black circles, *n* = 17 cells, 8 mice; two-way RM ANOVA, interaction genotype x stimulus *p* < 0.01, post hoc Holm–Sidak method *p* < 0.05 in the 50–90 μA range). Values are expressed as mean ± s.e.m. percentage change relative to their average maximal amplitude. (**B**) Maximal response area was not significantly different between the two groups (*t*-test, *p* = 0.223). (**C**) Representative recordings of excitatory postsynaptic currents (EPSCs, recorded at the chloride-reversal potential) and inhibitory postsynaptic currents (IPSCs, recorded at the AMPA/NMDA-reversal potential) from pyramidal neurons in slices from P60 wt and rd10 mice. (**D**) The average charge of excitation was not detected in rd10 mutant mice (*n* = 17 cells, *n* = 11 mice for wt and *n* = 14 cells, *n* = 12 mice for rd10 animals; Mann–Whitney rank sum test, *p* = 0.565). (**E**) The average inhibitory drive recorded from the same cells was significantly increased in the rd10 group compared to wt controls (*t*-test, *p* < 0.05). Data are expressed as mean ± s.e.m. * *p* < 0.05 and ns: not significant.

Spontaneous miniature EPSCs (mEPSCs) and IPSCs (mIPSCs) were recorded by whole-cell recordings targeted to layer II/III pyramidal cells of visual cortex slices of P60 wt and rd10 mice. No difference was present in cell input resistance (185.00 ± 42.89 MΏ in wt mice and 219.00 ± 49.81 MΏ in rd10 mice) and spike threshold (−54.18 ± 2.16 mV in wt mice and −54.07 ± 6.02 mV in rd10 transgenic mice; *n* = 11 cells for both groups). mEPSCs (Figure 3A,B,D) and mIPSCs (Figure 3E,F,H) from rd10 neurons had a mean frequency and instantaneous frequency similar to that recorded from wt mice. Although cumulative probability histograms of interevent intervals (IEI) revealed a difference between wt and rd10 EPSCs/IPSCs, the mean IEI and corresponding event frequency did not significantly differ between the two conditions (Figure 3C,G). Then, we determined synaptic currents in layer II/III pyramidal neurons evoked by layer IV stimulation. I/O curve was significantly shifted leftwards in rd10 neurons voltage clamped at −82 mV (Figure 4A), while the maximal amplitude of response was comparable between the two groups (Figure 4B). Because both excitatory and inhibitory current were depolarizing at the holding potential, this result is in agreement with the data from fEPSP experiment at P60 [33] as the same current produces a decrease in the excitability of the cell. To isolate excitatory and inhibitory currents within the same cell, we recorded EPSCs by holding the cell membrane at the measured chloride-reversal potential (E_Cl_ = −44.5 ± 3.3 mV in wt mice and E_Cl_ = −43.0 ± 5.2 mV in rd10 transgenic mice; *n* = 10 cells for both groups) and IPSCs at the measured reversal potential for postsynaptic AMPA and NMDA receptors (−2.0 ± 4.4 mV in wt mice, *n* = 11 cells, and 1.5 ± 4.3 mV in rd10 transgenic mice, *n* = 10 cells). Total excitatory and inhibitory drive was calculated by normalizing to the area response to the same stimulus and measured holding the cell at −82 mV. We found that the mean inhibitory synaptic charge was significantly increased in neurons of mutant mice, with no parallel change in excitatory currents (Figure 4C–E), producing a net shift in the balance between excitation and inhibition.

### 2.3. Thalamic Synaptic Processing in RP Animals

**Figure 5:**
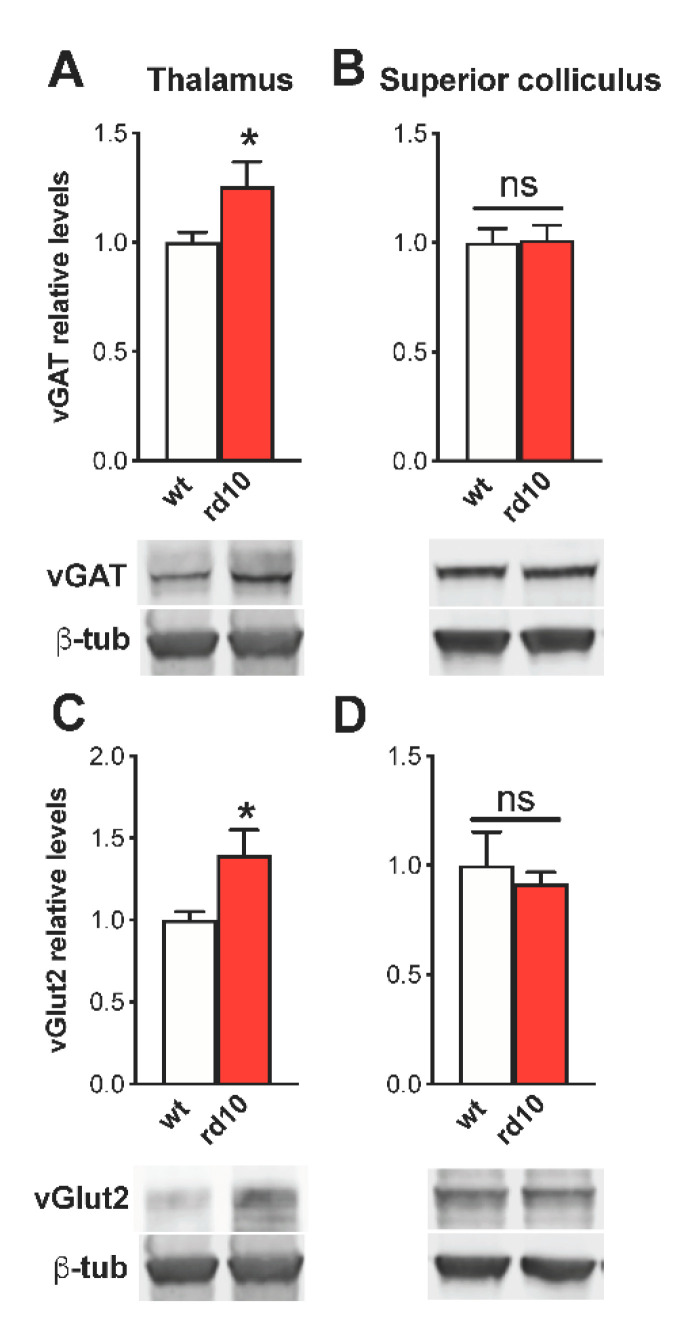
Alterations of vGAT and vGlut2 levels in the lateral geniculate nucleus (LGN) of rd10 animals at P60. In all panels, the inset is a representative immunoblotting showing the corresponding protein levels in the visual cortex of wild-type (wt) and rd10 transgenic animals. α-Tubulin (α-tub) is the internal standard. Diagrams show the percentage value of protein expression in relation to the control (wt) group. (**A**–**C**) vGAT and vGlut2 levels were larger in LGN of rd10 mice at P60 (*n* = 7) with respect to age-matched wt animals (*n* = 8, *t*-test, *p* < 0.05 for both comparisons). (**B**–**D**) No difference was present in the superior colliculus (SC) for either vGAT (*n* = 8 for both groups, *t*-test, *p* = 0.891) or vGlut2 (*p* = 0.611) expression. Animals used for protein quantification in LGN and SC are the same as those shown for visual cortex data. One sample was removed from the rd10 group for LGN analysis because of the undetectability of the vGAT band. Data are expressed as mean ± s.e.m. * *p* < 0.05 and ns: not significant.

To check whether abnormalities of synaptic transmission are spread widely to the whole visual system or restricted only to the cortex, we also measured levels of excitatory and inhibitory markers in the visual thalamus (lateral geniculate nucleus, LGN) and in the superior colliculus of rd10 animals at P60, corresponding to the stage at which the molecular alterations in the visual cortex were more evident. The expression of vGAT and vGlut-2 was significantly upregulated in the LGN of rd10 mice (Figure 5A,C), whereas no difference was detected in GAD65, GAD67, and vGlut-1 levels (Figure S4). In contrast, we did not find any change in the synaptic markers of superior colliculus (Figure 5B,D and Figure S4).

These results suggest that rd mutations affect the levels of excitation and inhibition not only in the visual cortex but also in the thalamus, which plays a central role in cortical functioning [42].

### 2.4. Lowering Inhibition in the Visual Cortex of rd10 Animals Hampers Visual Acuity

**Figure 6:**
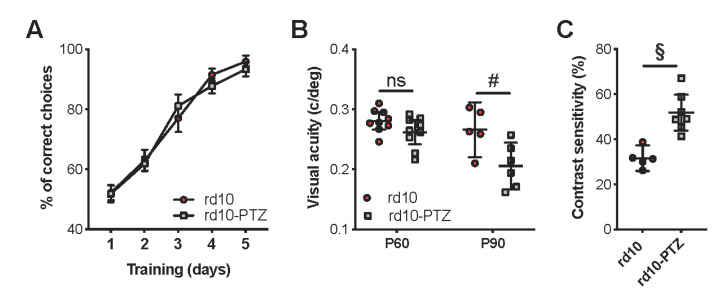
Antagonizing GABA neurotransmission in rd10 adult mice deteriorated visual acuity and contrast sensitivity. (**A**) Percentage of correct choices during the training phase of the visual water task in rd10 animals treated with the vehicle solution (red circles; rd10) or the PTZ drug (grey squares; rd10-PTZ). Three sessions of 10 trials were performed every day. The learning timeline of the two experimental groups was totally comparable (two-way RM ANOVA, treatment factor *p* = 0.761). Data are expressed as mean ± s.e.m. (**B**) Behavioral visual acuity of rd10 and rd10-PTZ animals plotted for age groups. A two-way ANOVA showed a significant effect of age and treatment (*p* < 0.01). Post-hoc Holm–Sidak test revealed a significant difference at P90 (rd10, *n* = 5 and rd10-PTZ, *n* = 6; *p* < 0.01), whereas PTZ did not significantly affect visual acuity at P60 (rd10, *n* = 9 and rd10-PTZ, *n* = 9; *p* = 0.313). Symbols represent single data values, and black lines indicate mean with 95% CI. (**C**) Contrast sensitivity significantly declined with PTZ treatment in rd10 mice at P90 (rd10, *n* = 5 and rd10-PTZ, *n* = 7; *t*-test, *p* < 0.001). Symbols represent single data values, and black lines indicate mean with 95% CI. # *p* < 0.01, § *p* < 0.001, and ns: not significant.

To test whether the change in inhibition/excitation balance in RP was associated with an alteration of visual functions, we treated a different group of rd10 mice with the noncompetitive GABAA antagonist pentylenetetrazole (PTZ) using a nonepileptic dose that can be safely administered to rodents for up to one year [43,44]. We observed that PTZ-treated mice had a significantly lower visual acuity and contrast sensitivity than age-matched vehicle-treated animals (Figure 6). These results indicated that the altered inhibition/excitation balance observed in the visual cortex of rd10 animals might be viewed as a compensatory attempt to increase the signal-to-noise ratio of the degraded sensory input originating from the degenerating retina.

## 3. Discussion

Most of the studies in the RP field addressed retinal structure and function, with very little attention to the visual cortex. Optical imaging and electrophysiological recordings have been commonly used to evaluate the potential of different therapeutic strategies to restore visually evoked responses in the cortex of RP models [7,8,9,10]. Only recently, a specific decay of cortical function in RP has been suggested, with loss of spatial and temporal responsiveness to visual stimuli [31,45] and waveform alterations of activity [28,29]. However, the contribution of cortical components to the pathogenesis of RP is still unexplored.

In the attempt to understand whether the progressive degeneration of retinal signals leads to an alteration in the visual cortical capability to elaborate sensory inputs, we investigated the effect of photoreceptor degeneration on intrinsic activity of cortical circuits in rd10 mice. Exploitation of a well-known mouse model of the disease allowed us to take advantage of ex vivo strategies to test visual cortex physiology without the confounding effects due to concomitant signals originating from the degenerating retina.

We demonstrated that rd10 mice showed an alteration of the excitatory/inhibitory balance in the lateral geniculate nucleus and the visual cortex at P60, leading to a net shift towards increased inhibition in local circuits. These changes in post-retinal information processing may represent a compensatory increase of the signal-to-noise ratio and improve the decoding of degraded inputs originating from the aberrant activity of retinal ganglion cells. Accordingly, we found that a non-epileptic dose of the GABA_A_ antagonist PTZ decreased visual acuity and contrast sensitivity of treated rd10 mice. In agreement with these results, data on patients showed that visual thresholds and visual field maps are strongly correlated to the deficits recorded by multifocal cortical potentials and not to those detected by retinal recordings [46].

Normal ocular dominance plasticity recently reported in the visual cortex of rd10 mice and RP patients [32,33] might seem at odds with the heightened inhibitory tone uncovered in visual cortical circuits. Given the high diversity of GABAergic interneuron subsets in the cerebral cortex, we can hypothesize that the control of information flow at circuit level and the regulation of neural plasticity in adult V1 of rd10 mice are mediated by distinct interneuron types, such as parvalbumin (PV)- and somatostatin (SST)-expressing cells [47]. Accordingly, PV-positive interneurons provide the main source of somatic inhibition [48] and whole-cell recordings are more sensitive to events affecting proximal dendrites and somata of pyramidal targeted neurons.

The signaling pathways linking photoreceptor degeneration with a reconditioned excitatory/inhibitory balance in the visual system remain obscure. Aberrant retinal activity might be a strong determinant of the anomalous calibration and functional reorganization of cortical circuits. Total lack of experience-driven electrical activity (dark rearing) delays the maturation of visual functions [49,50,51,52] and can induce significant rearrangements of synapses, including thalamocortical synapses, in the adult visual system [53,54]. Thus, it is possible to conceive that the deterioration of inputs from retinal ganglion cells in RP might result in a progressive change in neuronal selectivity and responsiveness in the visual cortex, possibly mediated by changes at the excitatory/inhibitory balance level. Consistently, lower levels of NGF and BDNF have been found in the retina, geniculate nucleus, and visual cortex of rats with RP [30]. An alternative, but not mutually exclusive, hypothesis is that retinal inflammation alters the cortical excitation/inhibition balance, acting through a downregulation of Otx2 expression [55]. Otx2 has been identified as a molecular retinocortical messenger regulating plasticity in the visual cortex, with a preferential action on inhibitory interneurons [56,57], and neuroinflammation at the retinal level in RP [58,59] might disrupt Otx2 maintenance of cortical circuits. Intriguingly, an opposite effect was found in the developing and adult visual cortex, where reducing cortical Otx2 resulted in a modification of the epigenetic state of parvalbumin cells facilitating cellular plasticity processes [60].

In summary, our results demonstrate that the visual cortex undergoes marked changes in intrinsic information processing alongside photoreceptor degeneration in RP, aimed at filtering noisy signals and increasing residual visual sensitivity. Current and future studies aimed at restoring vision in RP patients should not be limited to the preservation of retinal function but should also include a careful analysis of parallel cortical changes.

## 4. Material and Methods

### 4.1. Animals

We employed rd10 mutants (B6.CXB1-Pde6brd10/J on a C57Bl6J background; The Jackson Laboratory, Available URL: https://www.jax.org/strain/004297 (Accessed online: 28 August 2021; [34] RRID IMSR_JAX:004297) and wild-type mice (wt, C57BL/6J) purchased from The Jackson Laboratory. Females and males were uniformly distributed in the two experimental groups. Mice lived at 22 °C under a 12 h light–dark cycle (average illumination levels of 1.2 cd/m^2^). Food (4RF25 GLP Certificate, Mucedola, Settimo Milanese, Italy) and water were provided ad libitum. All experiments were carried out in accordance with the European Communities Council Directive of 22 September 2010 and were approved by the Italian Ministry of Health (authorization number 358/2015-PR).

### 4.2. Western Blot

To prevent circadian effects, animals were sacrificed within the same time frame (10:00–12:00 h; light phase). Brains were removed, and the visual cortex, superior colliculus, and lateral geniculate nucleus were dissected and frozen on dry ice. We compared wt and rd10 transgenic mice at P60 (*n* = 8 for both experimental groups), P120 (*n* = 8 for both groups), and P180 (*n* = 8 rd10 mice and *n* = 4 wt animals). The same animals were used to test the levels of presynaptic (vGAT, GAD65/67, vGlut1, and vGlut2) and postsynaptic proteins (gephyrin and PSD95) in the visual cortex. At P60, the same mice were used to measure the levels of vGAT, GAD65/67, vGlut1, and vGlut2 in the superior colliculus and the lateral geniculate nucleus. Unboiled protein extracts (15 μg) were separated by electrophoresis using 4–12% Criterion Tris-HCl Precast gels (BioRad, Segrate, Italy) and blotted; filters were blocked and incubated overnight at 4 °C with primary antibodies (anti-vGAT, 1:1000 dilution, Synaptic Systems, Göttingen, Germany, RRID AB_887872; anti-GAD65/67, 1:5000 dilution, Sigma-Aldrich RRID AB_477019; anti-vGlut1, 1:5000, Synaptic Systems RRID AB_887880; anti-vGlut2, 1:500, Synaptic Systems RRID AB_2285905; anti-gephyrin, 1:1000, Synaptic Systems RRID AB_887717; and anti-PDS95, 1:1000, Synaptic Systems RRID AB_2725761). Filters were probed with anti-β-tubulin antibody (1:15,000 dilution, Abcam, Cambridge, UK) as an internal standard. Blots were then incubated in infrared labeled secondary antibodies IRDye 700CW or 800CW (1:20,000 dilution, Li-Cor Biosciences, Lincoln, NE, USA). Filters were scanned using an Odyssey^®^ IR scanner (Li-Cor), and densitometry analysis was performed with Image Studio Lite software 4.0.

### 4.3. Analysis of Neurotransmitter Release in Visual Cortex Synaptosomes

Visual cortex was rapidly removed and synaptosomes were prepared as previously described [61]. We compared wt and rd10 transgenic mice at P60 (*n* = 9 for both experimental groups) and P180 (*n* = 7 for both groups). Briefly, after tissue homogenization in 14 volumes of 0.32 M sucrose, synaptosomes were purified by a discontinuous Percoll gradient. Purified synaptosomes were incubated (15 min, 37 °C) in the presence of 0.05 μM [^3^H]D-Asp, a nonmetabolizable analogue of glutamate [62,63], which labels the intraterminal releasable pools of the excitatory amino acid, or 0.1 μM [^3^H]GABA to label GABAergic axon terminals. Synaptosome aliquots were distributed on microporous filters placed at the bottom of a set of 24 parallel superfusion chambers maintained at 37 °C (Superfusion System, Ugo Basile, Comerio, Varese, Italy). Superfusion was started with a physiological medium at a rate of 0.5 mL/min and continued for 48 min [64]. After 39 min of superfusion to equilibrate the system, stimulation with a 90 s pulse of either 9 or 15 mM KCl was applied, with KCl substituting for an equimolar concentration of NaCl. When appropriate, Ca^2+^ was omitted from the superfusion medium at t = 20 min. Two 3 min samples (t = 36–39 and 45–48 min; basal release) and one 6 min sample (t = 39–45 min; stimulus-evoked release) were collected. Samples and superfused synaptosomes were counted for radioactivity. Tritium released in each sample was calculated as fractional rate × 100 (percentage of the total synaptosomal tritium content at the beginning of the respective collection period). The stimulus-evoked overflow was estimated by subtracting the transmitter content in the two 3 min fractions, representing the basal release, from the 6 min fraction collected during and after the stimulation pulse.

### 4.4. In Vitro Electrophysiology

Brains were removed and put in ice-cold cutting solution containing (in mM) KCl 2.5, NaHPO 41.25, NaHCO_3_ 15, HEPES 10, CaCl_2_ 0.5, MgSO_4_ 10, sucrose 230, glucose 16, myo-inositol 3, Na-pyruvate 3, and Na-ascorbate 0.4 (pH 7.3, 290 mOsm). Coronal slices of the visual cortex (250 μm thick) were obtained using a vibratome (Leica Microsystems, Buccinasco, Italy). Slices were allowed to equilibrate for at least 1 h in artificial cerebrospinal fluid (ACSF) containing (in mM) NaCl 119, KCl 2.5, NaHPO_4_ 1.25, NaHCO_3_ 15, HEPES 10, glucose 12.5, CaCl_2_·4H_2_O_2_, and MgSO_4_·7H_2_O_2_ (pH 7.3, 295 mOsm) at 35 °C before being moved to the recording chamber and perfused at a rate of 2 mL/min with oxygenated ACSF at 31 °C. Recordings were obtained from visually identified pyramidal cells of layer II/III (60× magnification, Zeiss lens) in whole-cell configuration. Borosilicate pipette electrodes (2.5–4.5 MΩ) with internal and external diameters of 0.86 and 1.5 mm, respectively (World Precision Instruments, Sarasota, FL, USA), were pulled using a P-97 (Sutter Instruments, Novato, CA, USA). Data were acquired using a Multiclamp 700A amplifier (Molecular Devices, San José, CA, USA) controlled by Clampex 8.2 via a Digidata 1322A (Molecular Devices). Data were sampled at 10 kHz and low-pass filtered at 1 kHz. Spontaneous mini excitatory (mEPSCs) and inhibitory (mIPSCs) postsynaptic currents were recorded holding the cells at −85 and −75 mV, respectively. For mESPC recordings, we added bicuculline methiodide (20 µM) and TTX (1 µM) to the perfusing ACSF. Pipette internal solution contained (in mM) potassium gluconate 130, HEPES 10, EGTA 1, CaCl_2_ 0.3, MgCl_2_ 1, ATP 4, GTP 0.3, and phosphocreatine 5 (pH 7.3, 285 mOsm). To isolate mIPSCs, we added 10 µM DNQX, 50 µM APV, and 1 µM TTX to ACSF, and 120 mM of potassium gluconate in the pipette solution was substituted for 120 mM KCl. Mini postsynaptic currents were analyzed using Minianalysis 6.0.7 software. Fluctuations of the current were detected as events when their amplitudes were higher than five times the standard deviation of the baseline. For each event, we evaluated the area, amplitude, interevent interval (IEI), and instantaneous frequency (IF). We compared wt (*n* = 6 for both mEPSC and mIPSC analysis) and rd10 transgenic mice (*n* = 7 for mEPSCs and *n* = 6 for mIPSCs) at P60. Evoked excitatory and inhibitory currents were measured in the same cells by voltage clamping the membrane potential at the reversal potential of one of the two postsynaptic currents, previously measured in different cells with excitatory (20 µM bicuculline methiodide in perfusion) or inhibitory (10 µM DNQX and 50 µM APV) components isolated by clamping the membrane at different potentials in 5 mV increments. Electrical stimulation (1 ms duration, maximum frequency 0.05 Hz) was delivered with a bipolar concentric stimulating electrode (FHC) placed in the middle of the layer IV. Whole-cell configuration was achieved using the following pipette solution (in mM): cesium methansulfonate 120, NaCl 8, ECTA 2, HEPES 10, Qx-314 5, Mg-ATP 2, Na-GTP 0.5, and Na-phosphocreatine 10 (pH 7.4, 280 mOsm). Evoked responses were analyzed using Clampfit 10.2. The average of 7 recorded traces was used to calculate the response amplitude and area with respect to the baseline. Excitatory and inhibitory responses were standardized to the response evoked at −82 mV in the same cell. Recordings were performed by an access resistance less than 30 MΩ and a membrane resistance of at least 150 MΩ. For all recordings, the liquid junction potential was calculated and adjusted a posteriori. Excitatory and inhibitory evoked responses were compared for wt (*n* = 11) and rd10 transgenic mice (*n* = 12) at P60. In a subset of the same animals (*n* = 8 for wt and *n* = 9 for rd10 mice), we characterized input–output curves.

### 4.5. Drug Administration

Pentylenetetrazole (PTZ) was purchased from Sigma Aldrich and dissolved in 1% low-fat chocolate milk. Animals were presented with 1% low-fat chocolate milk (vehicle treatment) or 1% low-fat chocolate milk–PTZ cocktail (in a volume of 5 mL/kg). PTZ was administered daily at 3 mg/kg over the course of 3 weeks. The treatment started at postnatal day (P) 40 for the measurement of visual functions at P60 and at P70 for P90 assessment. For drug delivery, mice were conditioned to drinking chocolate milk in their home cages from small Petri dishes. Then, animals were presented with small caps of milk–PTZ cocktail. Milk consumption typically occurred in ~5 min [43,65].

### 4.6. Behavioral Assessment of Visual Acuity and Contrast Sensitivity

Behavioral assessment of visual acuity was performed as previously described [36,37]. During the training phase, mice were conditioned to distinguish a low spatial frequency (SF, 0.116 c/deg) from homogeneous gray on a pseudorandom schedule. When the performance of animals was near perfect (80% or more) over 60 trials, incremental changes in the SF of the stimulus were made between in successive trials until the ability of animals to distinguish grating from gray fell to chance. In a different group of mice, contrast sensitivity was measured with the same procedure by progressively decreasing the contrast of the grating with SF of 0.116 c/deg. Visual acuity/contrast threshold was taken as the value corresponding to 70% correct choices on the sigmoidal function fitting the psychometric function, in which the percentage of correct choices was plotted against the SF/contrast of the grating. At P60 and P90, we compared the visual acuity of rd10 animals treated with the vehicle solution (rd10, *n* = 9 for P60 and *n* = 5 for P90) and rd10 mice administered with PTZ for 3 weeks (rd10-PTZ, *n* = 9 for P60 and *n* = 6 for P90). To avoid possible confounding effects of repeated pharmacological treatments, we used separate groups of animals for P60 and P90 measurements. At P90, we also assessed contrast sensitivity in an additional group of mice (*n* = 5 for rd10 and *n* = 7 for rd10-PTZ).

### 4.7. Statistical Analysis

All statistical analyses were performed using GraphPad Prism 8. Differences between two groups were evaluated with a two-tailed *t*-test. The significance of factorial effects was assessed with ANOVA/RM ANOVA followed by post hoc Holm–Sidak/Bonferroni test. Kolmogorov–Smirnov test was used to compare cumulative distributions of two datasets. Rank transformation was exploited for data not normally distributed. Level of significance was taken as *p* < 0.05.

## Data Availability

The datasets generated during the current study are available from the corresponding author on reasonable request.

## Supplementary Materials

The following are available online at https://www.mdpi.com/article/10.3390/ijms22179412/s1.
