# Supplementary material for: Visual Cortex Engagement in Retinitis Pigmentosa
Source: Int J Mol Sci. 2021 Aug 30;22(17):9412. doi: 10.3390/ijms22179412 (PMC8431500; doi:10.3390/ijms22179412)
Supplement: Supplementary file 1 [file ijms-22-09412-s001.zip › ijms-1326926-supplementary.pdf]

Fig. S1

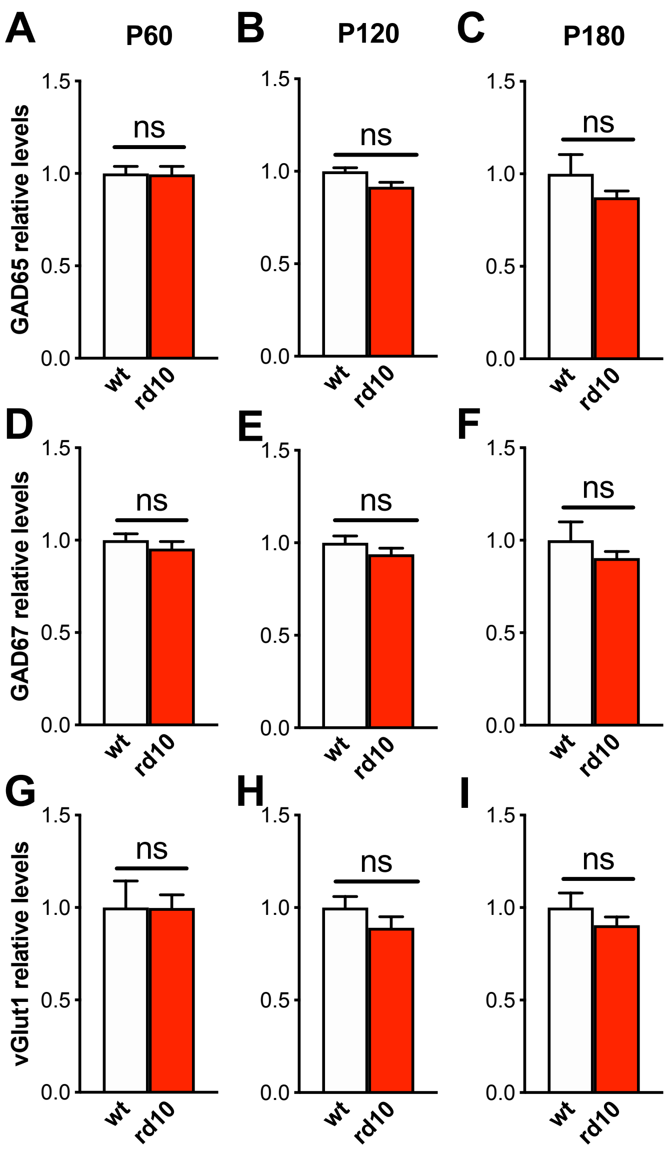

Figure S1. GABA synthesis and glutamatergic intracortical neurotransmission are not changed in the visual cortex of rd10 animals. Diagrams show the percentage value of protein expression in rd10 visual cortex in relation to the control (wt) group. No difference was present in GAD65 (P60: t-test p = 0.937, P120: t-test p = 0.110, P180: Mann-Whitney Rank Sum Test p = 0.461; **A-C**), GAD67 (P60: t-test p = 0.390, P120: t-test p = 0.217, P180: t-test p = 0.280; **D-F**) and vGlut1 (P60: t-test p = 0.992, P120: t-test p = 0.220, P180: t-test p = 0.283; **G-I**) protein expression at any age tested. These additional presynaptic markers have been tested in the same samples used for vGAT and vGlut2. Data are expressed as mean ± s.e.m. ns: not significant.

Fig. S2

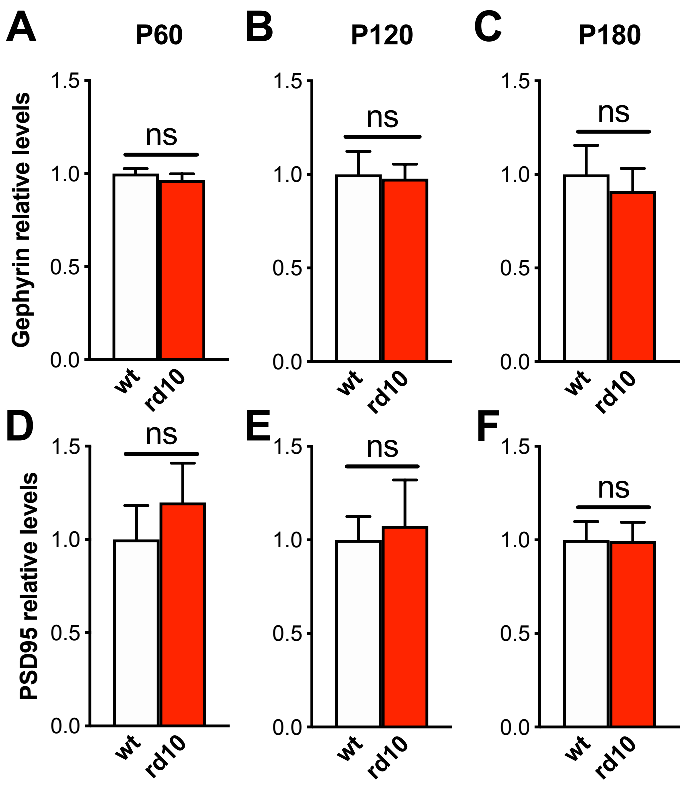

Figure S2. Regular expression of scaffold proteins at the postsynaptic density in the visual cortex of rd10 mice. Diagrams show the percentage value of protein amount in rd10 visual cortex in relation to the control (wt) group. No difference was present in gephyrin (P60: Mann-Whitney Rank Sum Test p = 0.234, P120: Mann-Whitney Rank Sum Test p = 0.798, P180: t-test p = 0.168; **A-C**) and PSD95 (P60: t-test p = 0.390, P120: t-test p = 0.217, P180: t-test p = 0.280; **D-F**) protein levels at any age tested. These postsynaptic markers have been tested in the same samples used for presynaptic proteins. Data are expressed as mean ± s.e.m. ns: not significant.

Fig. S3

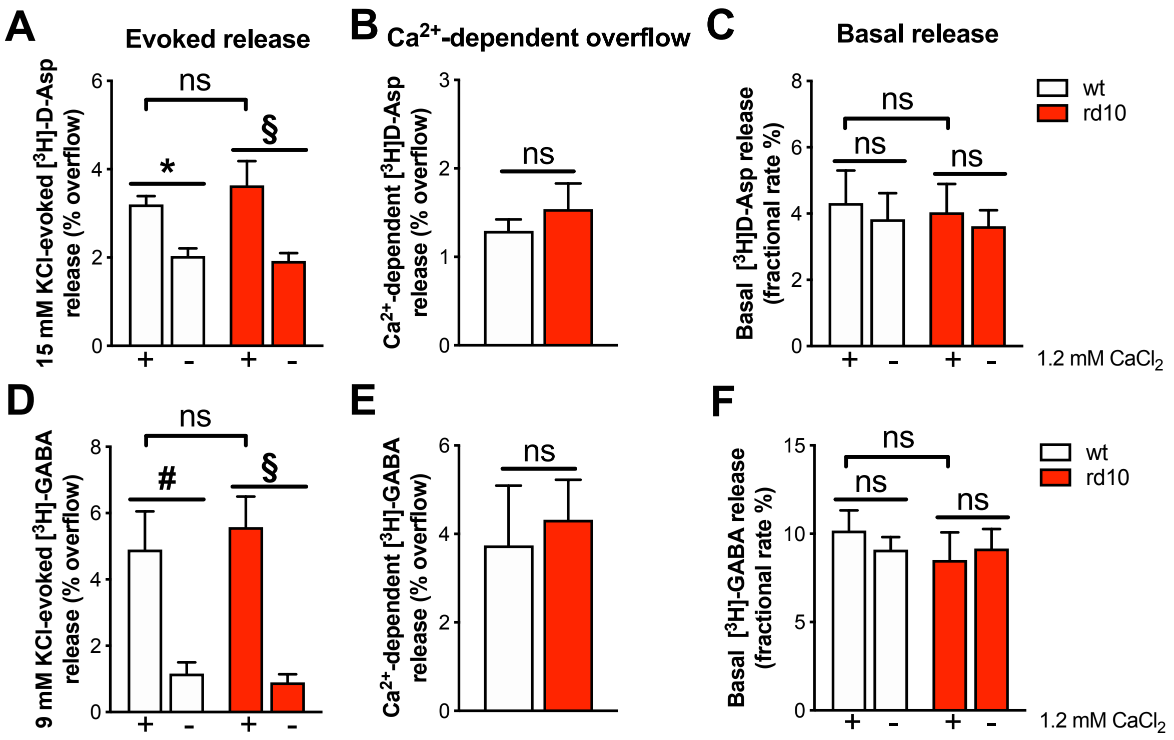

Figure S3. Release of glutamate and GABA from synaptosomes of rd10 visual cortex at P180. Synaptosomes were purified from the visual cortex of P180 wt and rd10 mice. Data are expressed as mean ± s.e.m. of 5-7 independent experiments run in triplicate. **(A)** 15 mM KCl-evoked [3H]D-Asp overflow was unchanged in the visual cortex of rd10 mice (Two-way RM ANOVA). Ca^{2+} lack reduces Asp release in both wt (Two-way RM ANOVA, post hoc Bonferroni test, $p < 0.05$) and rd10 animals ($p < 0.001$). **(B)** Ca^{2+}-dependent stimulus-evoked [3H]D-Asp overflow was not modified in rd10 visual cortex (t-test). **(C)** [3H]D-Asp basal release was not affected by rd10 mutation (Two-way RM ANOVA). **(D)** 9 mM KCl-evoked [3H]-GABA overflow was unaltered in the visual cortex of rd10 mice (Two-way RM ANOVA). Ca^{2+} removal strongly affects GABA release in both wt (Two-way RM ANOVA, post hoc Bonferroni test, $p < 0.01$) and rd10 animals ($p < 0.001$). **(E)** Ca^{2+}-dependent stimulus-evoked [3H]-GABA overflow was similar in wt and rd10 visual cortex (t-test). **(F)** [3H]-GABA basal release was not different in rd10 mice compared to wt (Two-way RM ANOVA). * $p < 0.05$, # $p < 0.01$, § $p < 0.001$, ns: not significant.

Fig. S4

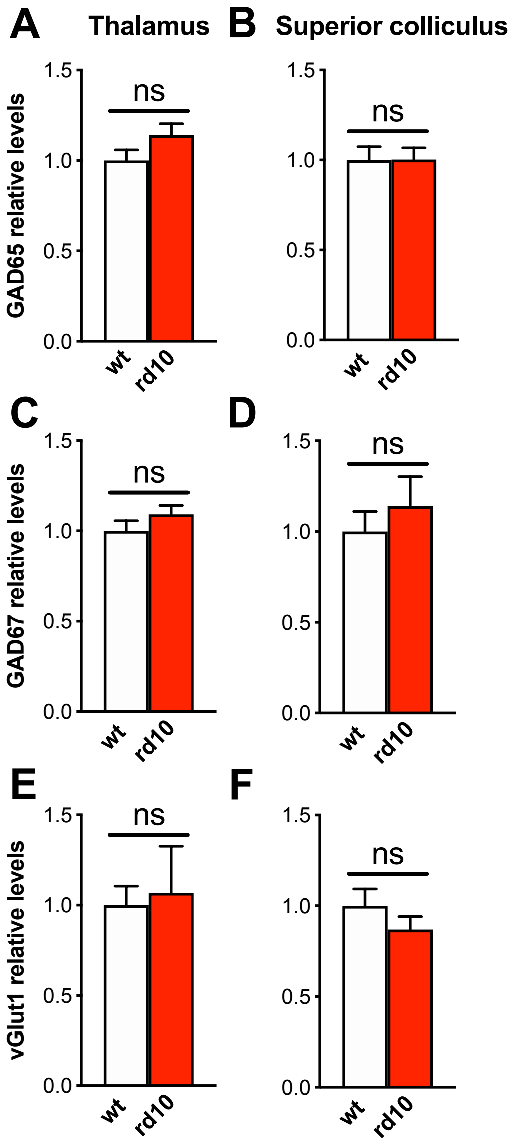

Figure S4. GABA synthesis and glutamatergic neurotransmission from the cortex are not changed in the lateral geniculate nucleus (LGN) and in the superior colliculus (SC) of rd10 animals at P60. Diagrams show the average percentage value of protein expression in rd10 thalamus and superior colliculus with respect to the control (wt) group. No difference was present in GAD65 (LGN: t-test p = 0.121, SC: t-test p = 0.979; **A,B**), GAD67 (LGN: t-test p = 0.248, SC: t-test p = 0.490; **C,D**) and vGlut1 (LGN: t-test p = 0.799, SC: t-test p = 0.284; **E,F**) protein expression at P60. These additional presynaptic markers have been tested in the same samples used for vGAT and vGlut2. Data are expressed as mean ± s.e.m. ns: not significant.
